# Minimally invasive vessel-preservation spleen preserving distal pancreatectomy-how I do it, tips and tricks and clinical results

**DOI:** 10.1007/s00464-023-10173-z

**Published:** 2023-06-23

**Authors:** Mohammad Abu Hilal, Lúcia Carvalho, Tess M. E. van Ramshorst, Marco Ramera

**Affiliations:** 1 Department of General Surgery, Istituto Ospedaliero Fondazione Poliambulanza, Brescia, Italy; 2 Department of Surgery, Centro Hospitalar de Entre O Douro E Vouga, Santa Maria da Feira, Portugal; 3 Department of Surgery, Amsterdam UMC, Location University of Amsterdam, Amsterdam, The Netherlands; 4 Cancer Center Amsterdam, Amsterdam, The Netherlands; 5 Department of Clinical and Experimental Sciences, University of Brescia, Brescia, Italy

**Keywords:** Distal pancreatectomy, Spleen-preserving distal pancreatectomy, Laparoscopic pancreatic surgery, Laparoscopic spleen-preserving distal pancreatectomy, Robotic surgery

## Abstract

**Background:**

Minimally invasive spleen-preserving distal pancreatectomy (SPDP) has emerged as a parenchyma-preserving approach and has become the standard treatment for pancreatic benign and low-grade malignant lesions. Nevertheless, minimally invasive SPDP is still technically challenging, especially when vessel preservation is intended. This study aims to describe the technique and outcomes of laparoscopic (LSPDP) and robot-assisted spleen-preserving distal pancreatectomy (RSPDP) with intended vessel preservation, highlighting the important tips and tricks to overcome technical obstacles and optimize surgical outcomes.

**Methods:**

A retrospective observational study of consecutive patients undergoing LSPDP and RSPDP with intended vessel preservation by a single surgeon in two different centers. A video demonstrating both surgical techniques is attached.

**Results:**

A total of 50 patients who underwent minimally invasive SPDP were included of which 88% underwent LSPDP and 12% RSPDP. Splenic vessels were preserved in 37 patients (74%) while a salvage vessel-resecting technique was performed in 13 patients (26%). The average surgery time was 178 ± 74 min for the vessel-preserving and 188 ± 57 for the vessel-resecting technique (p = 0.706) with an estimated blood loss of 100 mL in both groups (p = 0.663). The overall complication rate was 46% (n = 23) with major complications (Clavien Dindo ≥ III) observed in 14% (n = 7) of the patients. No conversions occurred. The median length of hospital stay was 4 days.

**Conclusion:**

This study presented the results after minimally invasive SPDP with intended vessel preservation by a highly experienced pancreatic surgeon. It provided tips and tricks to successfully accomplish a minimally invasive SPDP, which can contribute to quick patient rehabilitation and optimal postoperative results.

**Graphical Abstract:**

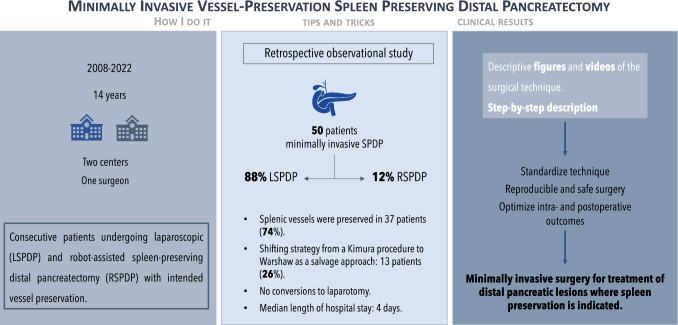

**Supplementary Information:**

The online version contains supplementary material available at 10.1007/s00464-023-10173-z.

Distal pancreatectomy is the term traditionally used for the resection of the tail and/or body of the pancreas, dividing the pancreas at any point to the left of the superior mesenteric vein-portal vein junction [1]. Traditionally, distal pancreatectomy also includes the spleen removal “splenectomy” due to the close relations between splenic vessels and the posterior pancreatic surface.

However, a splenectomy can be associated with different complications such as the overwhelming post-splenectomy infection syndrome [2] and thromboembolic events [3]. In addition, the immunity role of the spleen is thought to play a protective role in cancer development, with some epidemiologic studies showing an increased risk of overall malignancies after splenectomy [4–7]. Therefore, spleen-preserving distal pancreatectomy (SPDP) has become the preferred treatment for benign and low-grade malignancies in the pancreas [8].

Spleen preservation can be achieved by either preserving or sacrificing the splenic vessels. In the first technique, also named *Kimura* procedure [9, 10], a distal pancreatectomy is carried out while preserving the natural splenic perfusion through the splenic artery and vein. In the second technique, known as *Warshaw* procedure [11, 12], the splenic artery and vein are ligated, and the splenic perfusion becomes dependent on short gastric and left gastroepiploic vessels.

Over the last decades, minimally invasive approaches such as laparoscopic and robot-assisted surgery, have been successfully adopted in distal pancreatectomy, with laparoscopic distal pancreatectomy (LDP) currently being the most performed pancreatic laparoscopic surgery, particularly for benign and low-grade malignant lesions [13–15]. However, minimally invasive SPDP is a technically challenging procedure, especially when vessel preservation is intended. In our experience, a standardization of the surgical technique as well as the adoption of some specific tips and tricks are essential to facilitate the surgical delicate steps and ensure a safe completion of these complex procedures. We herein describe our technique and outcomes of laparoscopic SPDP (LSPDP) and robotic SPDP (RSPDP) with intended vessel preservation, highlighting the important tips and tricks to overcome the technical obstacles and optimize surgical outcomes. Two videos demonstrating both LSPDP and RSPDP procedures and some special delicate steps are embedded. Also, we discuss our shifting strategy from a Kimura procedure to Warshaw as a salvage approach, to avoid splenic preservation failure and conversion to open surgery, especially in the first phase of the learning curve.

## Materials and methods

### Study design

This is a retrospective cohort study of all LSPDP and RSPDP procedures performed by a single surgeon for benign or low-grade malignant lesions of the pancreas between 2008 and 2022 in two different hospitals; University Hospital Southampton (UHS: 01/2008–08/2019) and Fondazione Poliambulanza Istituto Ospedaliero (Poliambulanza: 08/2019–10/2022). The data of consecutive patients were prospectively collected in an anonymized database. Data collection included: patient demographics, perioperative details, and postoperative results. The study has been reviewed and approved by the ethical committee of Fondazione Poliambulanza with study reference number: NP 5795. The study followed the guidelines of the Strengthening the Reporting of Observational studies in Epidemiology (STROBE) [16].

### Perioperative management

All patients were studied preoperatively with cross-sectional imaging with multi-phase intravenous contrast computed tomography (CT) of the abdomen and pelvis. In some cases, contrast-enhanced magnetic resonance imaging (MRI) and endoscopic ultrasound (EUS) were used to provide further information on the lesion’s nature and its anatomic relationship to surrounding structures. EUS-guided fine needle aspiration (FNA) was performed when indicated.

For patients with suspicion of a neuroendocrine tumor, a dual tracer (^18^F-FDG and ^68^ Ga-DOTATOC) PET/CT, and a serum chromogranin A assay were also performed. Assessment of serum hormone concentrations was reserved for patients with hormonal symptoms.

The indication for a spleen-preserving distal pancreatectomy was discussed in a multidisciplinary setting and decided based on radiological and histological findings.

### Outcome measures

Demographics and tumor characteristics included age, sex, body mass index (BMI), tumor size, preoperative workup, and histopathologic analysis. Perioperative outcomes included operative time (OT), estimated blood loss, surgical approach, conversion to laparotomy, postoperative blood transfusion and the number of units, length of Intensive Care Unit (ICU) and hospital stay (LOS), pancreatic fistula [as defined by the International Study Group on Pancreatic Surgery (ISGPS)] [17, 18], reoperation, hospital readmission, 90-day morbidity, and 90-day mortality. Complications were assessed and classified according to the Clavien–Dindo classification [19, 20]. Major complications were classified as a Clavien-Dindo grade 3a or higher [20]. Postoperative pancreatic fistula (POPF) and post-pancreatectomy hemorrhage (PPH) were defined and classified according to the current definitions of the ISGPS [18, 21, 22].

### Statistical analysis

Statistical analysis was performed using IBM SPSS Statistics for Windows version 26.0 (IBM Corp., Orchard Road Armonk, New York, US). Comparative analyses were performed between the vessel-preserving and vessel-resecting groups, RSPDP and LSPDP groups, and time periods 2008–2014 and 2015–2022. Time periods were selected based on the surgeon’s experience and completion of the learning curve of minimally invasive distal pancreatectomy [23]. The Student t, Mann–Whitney U, Chi-Square, and Fisher’s exact test were used as appropriate. Categorical data are presented as proportions, continuous data as mean with standard deviations (SD), or median with interquartile range (IQR) based on its distribution. A two-sided p-value of < 0.05 was considered statistically significant.

## Operative technique

### Operative setting of pure LSPDP and RSPDP

In our experience, the main difference between LSPDP and RSPDP is the port position and the use of an ultrasonic dissector in the laparoscopic approach.

**Fig. 1:**
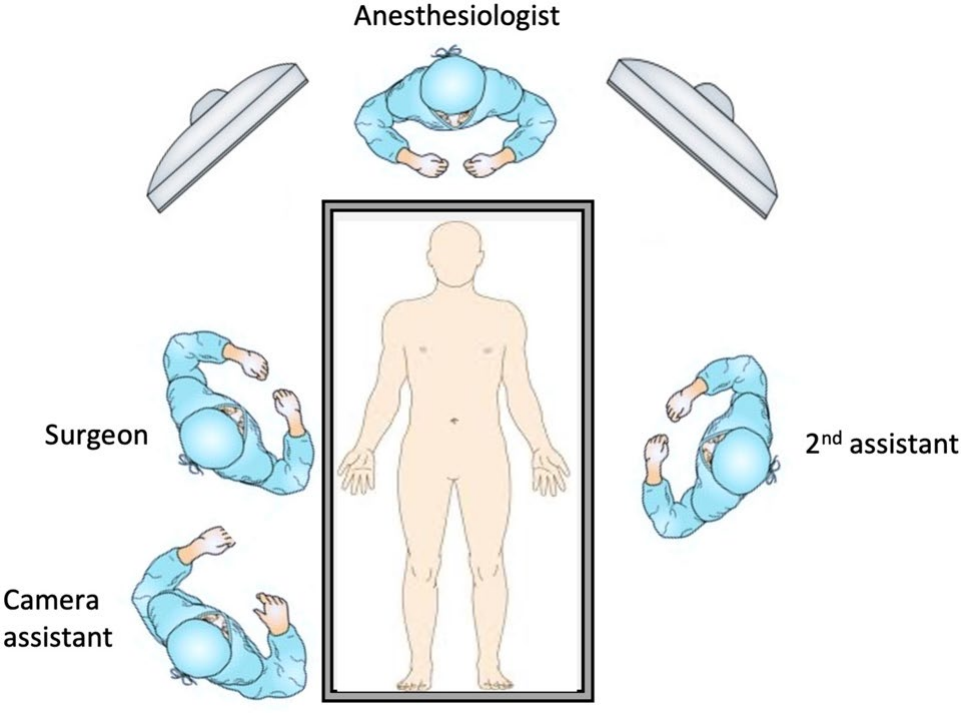
Position of the surgical team in the operating room.

In LSPDP, patients are positioned supine, in a reverse Trendelenburg, and right-tilt position. The right tilt ranges between 30 and 60° according to the location of the tumor—the more medial the lesion, the less tilt is needed to allow for better vision and access and vice versa. The legs are closed and the surgeon and camera assistant stand on the patient’s right side, while the second assistant stands on the patient's left (Fig. 1).

**Fig. 2:**
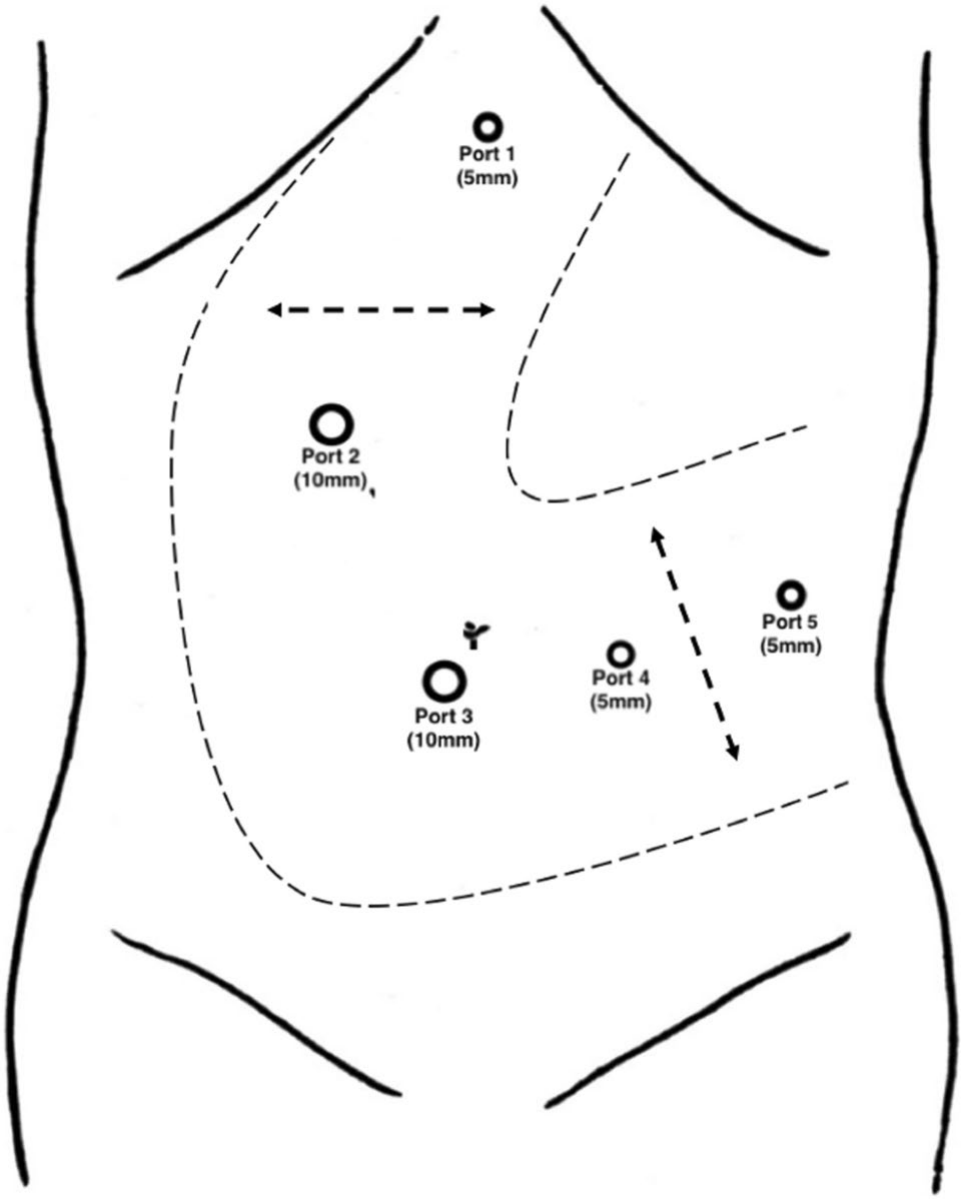
Trocar site placement for laparoscopic spleen-preserving distal pancreatectomy, with adaptations for patients with obesity and lesions in the distal part of the pancreatic tail or for small patients and pancreatic lesions medially located.

Four ports (two 12-mm ports and two 5-mm ports) are routinely used. The optical trocar of 12-mm is inserted paraumbilical and the pneumoperitoneum is established, 3 other trocars are positioned in a L shape curve (Fig. 2). The L curve may be moved to the left in case of a large or obese patient or when the lesion is in the distant part of the pancreatic tail. The L curve may be moved to the right in case of a small patient or a medially located lesion.

In RSPDP, the patient is placed in a supine position with legs apart and the assistant standing between the patient’s legs. The table is oriented in reverse Trendelenburg position of 25–30º and tilted to the patient’s right side (5º).

**Fig. 3:**
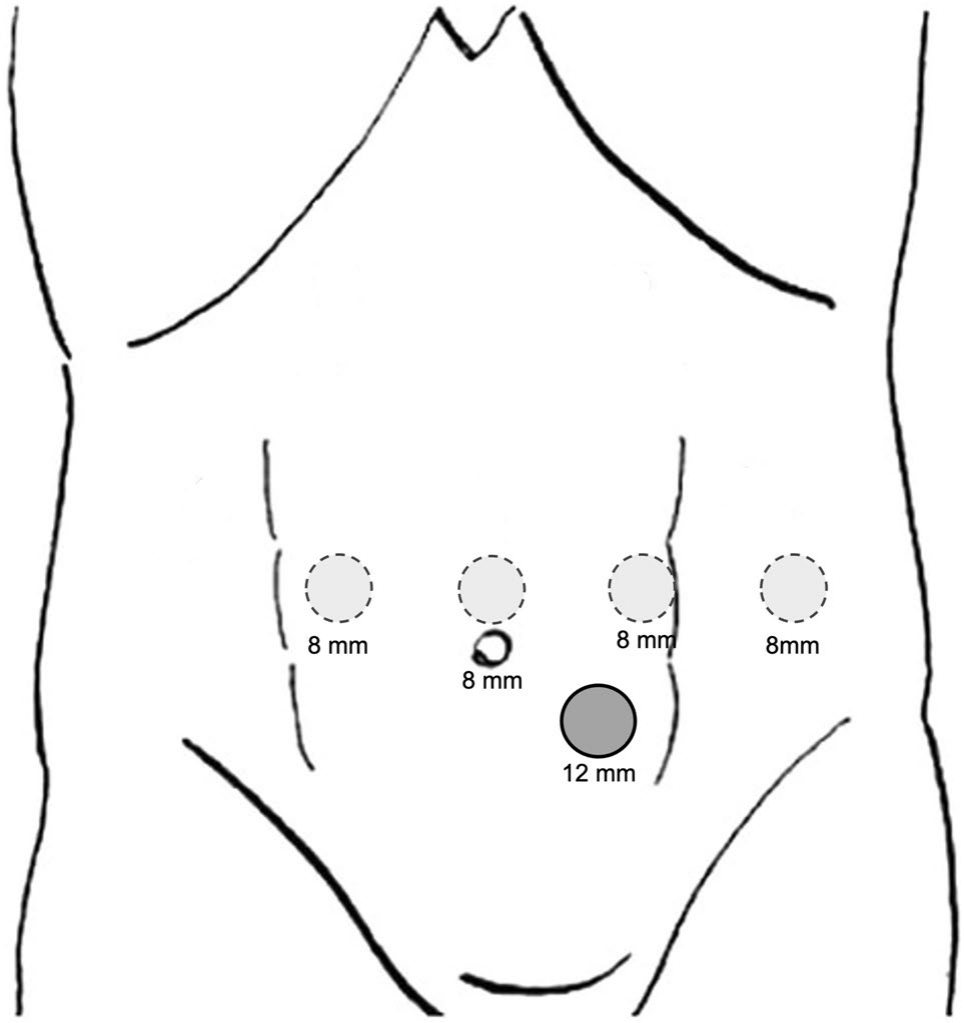
Trocar site placement for robotic spleen-preserving distal pancreatectomy.

A 12-mm laparoscopic trocar is inserted in the left pararectal region, about 5 cm below the straight transverse umbilical line of robotic trocars. Four robotic trocars are placed as shown in Fig. 3. In an obese patient, it may be necessary to place the robotic trocars along a line 3–4 cm above the umbilicus. Another optional 5-mm laparoscopic trocar can be placed in the right pararectal region, below the robotic trocar line.

*Tip*: It is important to remember that, except for specific operating tables, it is not possible to move the patient during the robotic procedure so it should be good practice to check the exposure of the target area before completing the docking.

### Operative steps

**Fig. 4:**
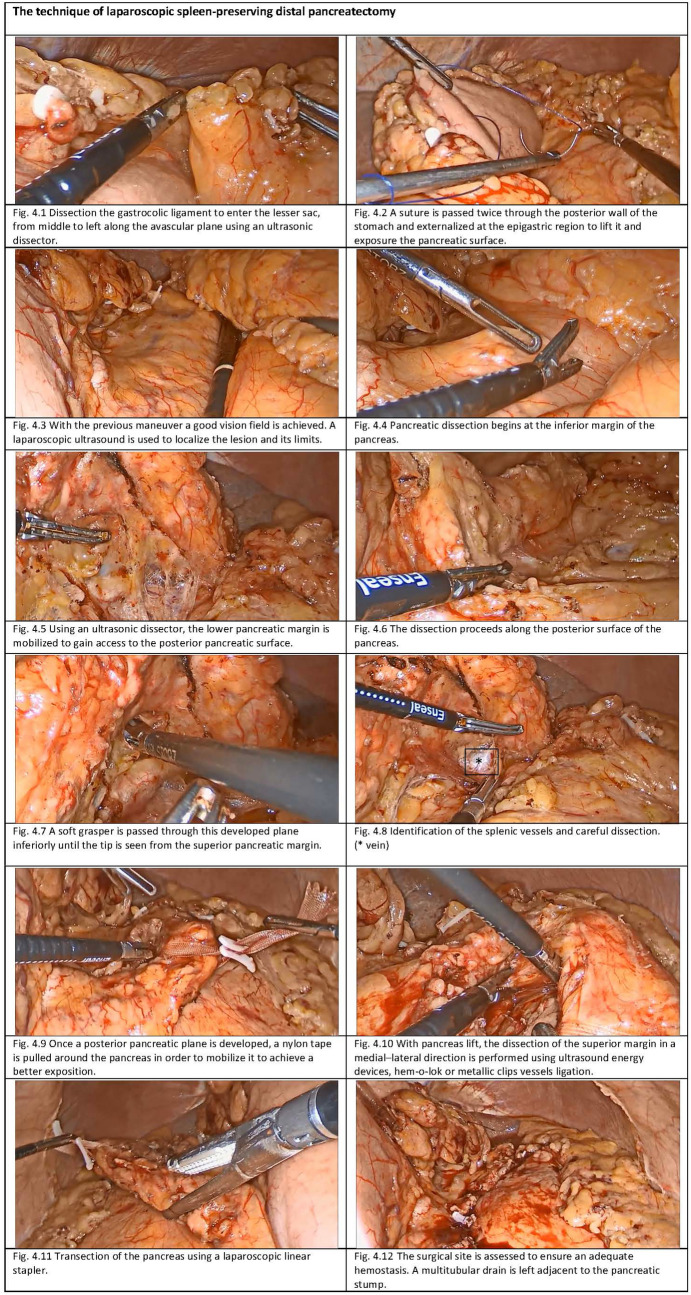
Operative steps of laparoscopic spleen-preserving distal pancreatectomy. 4.1: Dissection of the gastrocolic ligament to enter the lesser sac, from middle to left along the avascular plane using an ultrasonic dissector. 4.2: A suture is passed twice through the posterior wall of the stomach and externalized at the epigastric region to lift it and expose the pancreatic surface. 4.3: With the previous maneuver a good vision field is achieved. A laparoscopic ultrasound is used to localize the lesion and its limits. 4.4: Pancreatic dissection begins at the inferior margin of the pancreas. 4.5: Using an ultrasonic dissector, the lower pancreatic margin is mobilized to gain access to the posterior pancreatic surface. 4.6: The dissection proceeds along the posterior surface of the pancreas. 4.7: A soft grasper is passed through this developed plane inferiorly until the tip is seen from the superior pancreatic margin. 4.8: Identification of the splenic vessels and careful dissection. (* vein). 4.9: Once a posterior pancreatic plane is developed, a nylon tape is pulled around the pancreas in order to mobilize it to achieve a better exposition. 4.10: With the pancreas lifted, the dissection of the superior margin in a medial–lateral direction is performed using ultrasound energy devices, hem-o-lok or metallic clips vessels ligation. 4.11: Transection of the pancreas using a laparoscopic linear stapler. 4.12: The surgical site is assessed to ensure an adequate hemostasis. A multitubular drain is left adjacent to the pancreatic stump (Color figure online).

**Fig. 5:**
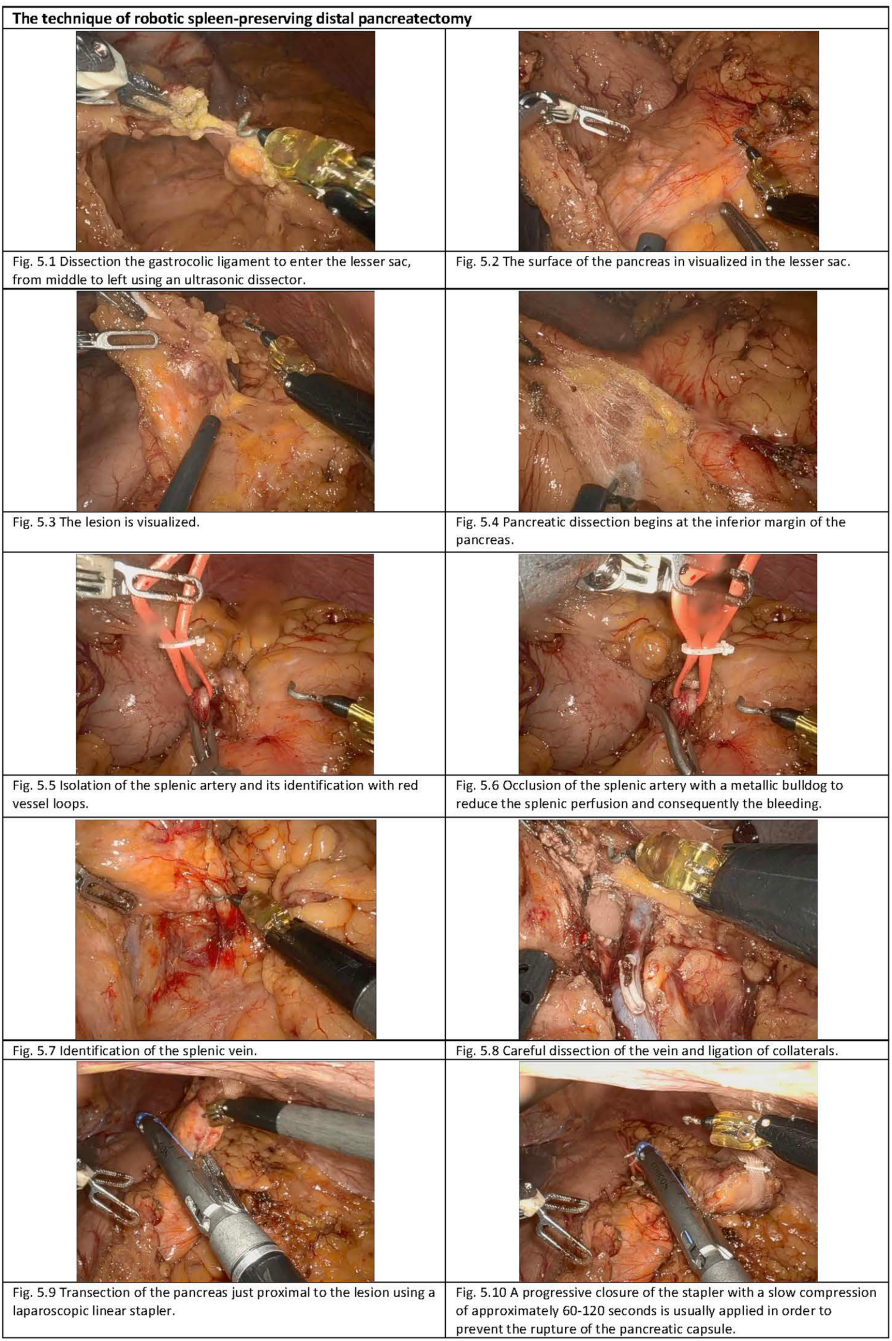
Operative steps of robotic-assisted spleen-preserving distal pancreatectomy. 5.1: Dissection of the gastrocolic ligament to enter the lesser sac, from middle to left using an ultrasonic dissector. 5.2: The surface of the pancreas in visualized in the lesser sac. 5.3: The lesion is visualized. 5.4: Pancreatic dissection begins at the inferior margin of the pancreas. 5.5: Isolation of the splenic artery and its identification with red vessel loops. 5.6: Occlusion of the splenic artery with a metallic bulldog to reduce the splenic perfusion and consequently the bleeding. 5.7: Identification of the splenic vein. 5.8: Careful dissection of the vein and ligation of collaterals. 5.9: Transection of the pancreas just proximal to the lesion using a laparoscopic linear stapler. 5.10: A progressive closure of the stapler with a slow compression of approximately 60–120s is usually applied in order to prevent the rupture of the pancreatic capsule (Color figure online).

The operative steps are not significantly different between LSPDP and RSPDP. The laparoscopic technique is shown in Fig. 4, and the robot-assisted technique in Fig. 5.

A careful assessment of the peritoneum and abdominal organs should be performed to confirm the preoperative staging and check for any contraindications.

The dissection starts by entering the lesser sac through the gastrocolic ligament and opening it broadly from middle to left following the avascular plane with an ultrasonic dissector until reaching the short gastric vessels, taking care to preserve them and the gastroepiploic arcade (Fig. 4.1; 5.1). During RSPDP, this phase is performed using only the monopolar hock and the bipolar forceps, as in the rest of the procedure.

Even if a vessel-preserving SPDP is intended, it is advised to try to keep the short gastric vessels if possible. This allows more anatomical vascular circulation and the possibility to shift to a *Warshaw* technique if vessel preservation is not possible.

The splenocolic ligament is then dissected up to the splenic flexure.

*Tips*

A. The splenic flexure should be well mobilized to permit the exposure of the pancreatic tail.
B. The stomach is cephalically retracted by passing a nonabsorbable monofilament nonabsorbable suture twice through the posterior gastric wall and externalizing it via the epigastric port, exposing the pancreatic surface (Fig. 4.2; 5.2). In RSPDP, the stitches can be externalized using an Endo Close™.

Intraoperative ultrasound is routinely performed to determine the tumor location and extension (Fig. 4.3; 5.3).

At this stage, the inferior border of the pancreas should be identified, and the dissection starts using an ultrasonic dissector by mobilizing the lower pancreatic margin and gaining access to the posterior pancreatic surface (Figs. 4.4, 4.5; 5.4).

*Tip* The inferior margin of the pancreas represents a good avascular plane for starting the dissection. Careful attention should be paid in pancreatic neuroendocrine tumor (PNET) cases where neovascularization is not uncommon and hypertrophied vessels can be encountered.

During the initial phases including the ultrasound assessment, it is a good practice to identify the splenic artery optimally at the most superficial, extra parenchymal point medial to the lesion (Fig. 5.5). When the splenic artery is identified, it should be dissected, slung, and occluded using a laparoscopic metallic bulldog clamp. This maneuver temporarily reduces the pancreatic and splenic perfusion, so the flow returns into the splenic vein, and easier vessel skeletonization can be achieved with less risk of bleeding during the dissection (Fig. 5.6).

*Tip* It is recommended to divide the sling into 4 equal pieces. These short segments are easy to manage and less disturbing in the surgical field and pass through the 5-mm port. Having a specific number of equal pieces can be very helpful during the final phase of counts and checks.

The dissection of the posterior pancreatic surface continues using a combination of a diathermy hook, and an ultrasonic dissector (in LSPDP) until the superior pancreatic margin is identified (Fig. 4.6). At this stage, a soft grasper or a Goldfinger is passed through this developed plane inferiorly until the tip is seen from the superior pancreatic margin. A nylon tape then is passed and used to encircle the pancreas and secured with a hem-o-lok clip allowing to hang the pancreas which then can be lifted to expose surgical planes (Fig. 4.7, 4.9). This hanging maneuver is very helpful to manipulate the pancreas without risking damaging the parenchyma.

In some cases, dissection along the superior pancreatic margin may be needed to facilitate the passage of the sling.

With the pancreas lifted, the pancreatic body and tail are further mobilized to permit a good proximal and distal exposure of the lesion.

At this stage, the splenic vein can be identified and carefully dissected to enable its slinging, using a diathermy hock and a right-angle forceps (Fig. 4.8).

*Tip* Special attention should be paid to avoid the injury of posterior pancreatic branches. If the diathermy is used it is a good practice to lower the power to a minimum to avoid incidental heat injuries to the vein. Once a safe passage is developed the vein is slung with a blue vessel loop.

With retraction on the arterial or the venous loop, dissection of the vessels from the pancreatic parenchyma, identifying and securing any pancreatic branches, is completed. When a salvage Warshaw procedure is needed, the vessels are ready to be controlled. Vein preparation is needed 2–3 mm proximally to the intended line of division. In case of a *Kimura* procedure, this should continue from that point till the extreme end of the pancreatic tail.

*Tips*

A. Slinging the splenic vessels allows not only for a better field exposure and an easier temporary positioning of a bulldog, but also ensures an efficient clipping of these vessels when needed.
B. Pulling on the slings should be done with great attention to avoid injuries of small pancreatic vessels, especially venous branches.
C. Avoid pulling the vein for long periods to prevent thrombosis.
D. Small pancreatic vessels should be carefully secured. This can be achieved using hem-o-loks clips or using an ultrasonic device. The first option is preferred if there is enough space to allow this. However, if the space is narrow, it is better to ensure good hemostasis using the hemostatic device instead of risking unsafe clipping attempts.

The dissection is then continued in a medial–lateral direction, using the same devices (Figs. 4.10; 5.7, and 5.8). If performing a *Warshaw* technique, the splenic artery and vein should be ligated at the splenic hilum as close as possible to the pancreas and proximally at the point of pancreatic transection.

The pancreas is divided, maintaining a clear margin from the lesion, using a laparoscopic linear stapler device (Fig. 4.11; 5.9). The stapler cartridge chosen should be adjusted to the thickness and texture of the pancreas. This decision must result from a balance between a gentle compression that does not fracture the pancreatic parenchyma and enough strength to perform a correct hemostasis.

*Tips*

A. Progressive closure of the stapler with slow compression of approximately 60–120s until complete closure can prevent rupture of the pancreatic capsule (Fig. 5.10).
B. It is not uncommon that the pancreas appears bulky due to adherences or overlapping between pancreatic acinar, in these cases if a smooth separation can be obtained easily; this may help in thinning the resection line thus achieving a better stapling with less risk of parenchyma fracturing. Also, if a pancreatic marginal vessel is noted it may be useful to ligate or clip it proximally. This does reduce the risk of stump bleed which can lead to parenchymal hematomas, an important risk factor for fistulas. This step is shown in Supplementary Video 1.
C. If any serious marginal bleeding occurs, it should be controlled with stitches, hem-o-lok or clips as appropriate. Minor bleeding can be controlled with bipolar or a piece of surgical snow.
D. It is important to achieve hemostasis but at the same time to be aware that suturing and manipulating the resection margin can by itself increase the risk of fistulas. Hence, a judicious management strategy of the margins is essential.

Any slings and bulldogs are removed from the count.

The specimen is removed through a Pfannenstiel incision using an impermeable extraction bag. The peritoneum and fascia are closed, visualized laparoscopically to ensure optimal closure.

Finally, the resection site is examined, the pancreatic stump is revised, and the hemostasis is ensured (Fig. 4.12). A combination of absorbable hemostatic materials can be applied.

*Tip* At the end of the surgery, spleen viability should be assessed based on its appearance. When in doubt, an ultrasound evaluation can be used to verify the spleen perfusion.

A multitubular drain is left at the surgical field adjacent to the pancreatic resection margin and externalized through the left 5 mm port.

The trocars are removed under direct vision and the fascia sheath of 10-mm or larger trocar port sites is sutured.

*Tip* If the lesion is too close to the splenic vessels, if the dissection is too difficult, or in cases of inadvertent damage of the splenic artery or vein, it is preferable to ligate the splenic vessels, converting the surgery into a *Warshaw* procedure or even to a distal pancreatectomy with splenectomy. In the same way, if even with additional trocars the dissection is not possible, converting to open surgery is always an option.

In cases where benign or low-malignant lesions are impossible to dissect from the splenic hilum, when intraoperatively the lesion seems malignant, when there are suspected splenic lymph nodes, or when there is deficient perfusion of the spleen, a splenectomy is recommended.

## Results

### Patient characteristics

In total, 214 patients underwent minimally invasive distal pancreatectomy during the study period (UHS: 184; Poliambulanza: 30). Of the 214 patients, 164 were excluded from this study as they underwent a planned splenectomy based on preoperative decision making. Preoperative contraindications for SPDP in these patients included malignant/borderline lesions or metastases to the tail, large benign (MCN) lesions (> 50 mm), location of the lesion nearby or in the splenic hilum, splenic vessel involvement, suspected lesions, unclear lesions on preoperative imaging, pancreatitis (patients that presented with pancreatitis and suspicious duct obstruction, patients that developed pancreatitis after EUS guided aspiration or biopsy, or patients with a history of chronic pancreatitis) and hesitance of splenic preservation in neuroendocrine tumors. Also, in the surgeon’s first 3–4 surgical years, no SPDP was performed for any indication as experience in distal pancreatectomy with splenectomy had yet to be gained. Eventually, 50 patients (33.1%) underwent intended SPDP during the study period (40 at UHS and 10 at Poliambulanza).

The median age of the SPDP cohort was 65 years and 24 patients (48%) were female. The median BMI was 25.8 (22.2–32.0) kg/m2. The diagnosis was incidental in 69.8% of the cases.

Indications for SPDP included neuroendocrine tumors, cystic lesions and intraductal papillary mucinous neoplasm (IPMN). The mean size of the lesion was 29 ± 17.8 mm. All patient characteristics are shown in Table 1.

**Table 1:** Patient demographics total cohort

	Overall (n = 50)
Age, y (IQR)	65 (42–72)
Female, n (%)	24 (48%)
BMI, Kg/m^2^ (IQR)	25.8 (22.2–32.0)
ASA score, n (%)	
1	8 (16%)
2	32 (64%)
3	10 (20%)
Incidental diagnosis, n (%)	30/43 (69.8%)
Pathology, n (%)	
Cystic lesion	21 (42%)
PNET	21 (42%)
Other	8 (16%)
Size, mm (mean ± Sd)	26.0 ± 14.8

*IQR* interquartile range; *BMI* body mass index; *IQR* interquartile range; *ASA* American Society of Anesthesiologists physical status classification; *PNET* pancreatic neuroendocrine tumour; *SD* standard deviation

### Operative details

Among the 50 patients who underwent SPDP, 40 (80%) underwent LSPDP and 10 (20%) RSPDP. In all SPDP procedures, a Kimura procedure was intended but eventually achieved in 37 patients (74%). A salvage Warshaw procedure was required in 13 (26%) patients. No conversion to laparotomy was required.

Overall, the mean OT was 185 ± 71 min and estimated blood loss 100 mL (IQR 50–262). The intraoperative information is shown in Table 2.

**Table 2:** Intraoperative outcomes total cohort

Overall (n = 50)	
Surgical Approach, n (%)	
- Laparoscopic	44 (88%)^#^
- Robotic	6 (12%)
Conversion to laparotomy rate, n (%)	0 (0%)
Vessel-preserving (Kimura) achieved	37 (74%)
Multivisceral resection, n (%)	7 (14%)
Cholecystectomy	5 (10%)
Left lateral sectionectomy	1 (2%)
Prostatectomy	1 (2%)
Operative time, min (± SD)	185 ± 71
Intraoperative blood loss, ML (IQR)	100 (50–262)
Intra- or post-operative blood transfusion, n (%)	1 (2%)

*SD* standard deviation; *IQR* interquartile range

^#^ One robotic approach converted to laparoscopy

### Vessel-preserving (Kimura) versus vessel-resecting (Warshaw)

Analyses of outcomes between the Kimura and Warshaw groups are displayed in Table 3. No differences in operative time and estimated blood loss were observed between the groups. The median LOS was 4 days in the Kimura versus 5 days in the Warshaw group (*p* = 0.047). The overall incidence of complications was 46%, of which 48.6% in the Kimura group and 38.5% in the Warshaw group (p = 0.747). Major complications were observed in 7 patients (14%), of which 6 in the Kimura group versus 1 in the Warshaw group (p = 0.660). Two patients (4%) were reoperated, all in the Kimura group, one with bleeding from the splenic vein, which was managed by a conversion to a Warshaw, and one with hemoperitoneum requiring an additional laparoscopic splenectomy.

**Table 3:** Comparison of outcomes between vessel-preserving and vessel-resecting technique

	Overall (n = 50)	Vessel Preserving (n = 37)	Vessel Resecting (n = 13)	*P*
Age, y (IQR)	65 (42–72)	65 (39–73)	63 (42–71)	0.973^**†**^
Female, n (%)	24 (48%)	18 (48.6%)	6 (46.1%)	0.877*
BMI, Kg/m^2^ (IQR)	25.8 (22.2–32.0)	29.0 (22.6–32.1)	21.1 (19.1–23.1)	0.105^**†**^
ASA score, n (%)				0.633*
1	8 (16%)	5 (13.5%)	3 (23%)	
2	32 (64%)	25 (67.6%)	7 (54%)	
3	10 (20%)	7 (18.9%)	3 (23%)	
Incidental diagnosis, n (%)	30/43 (69.8%)	23/32 (71.9%)	7/11 (63.6%)	0.709^§^
Pathology, n (%)				0.051*
Cystic lesion	21 (42%)	14 (37.8%)	7 (53.8%)	
PNET	21 (42%)	19 (51.4%)	2 (15.4%)	
Other	8 (16%)	4 (10.8%)	4 (30.8%)	
Size, mm (mean ± Sd)	26.0 ± 14.8	27.1 ± 15.6	22.9 ± 12.7	0.647^**†**^
Operative time, min (± SD)	185 ± 71	178 ± 74	188 ± 57	0.706^**†**^
Intraoperative blood loss, mL (IQR)	100 (50–262)	100 (50–275)	100 (100–250)	0.663^**†**^
Intra- or post-operative blood transfusion, n (%)	1 (2%)	1 (2.7%)	0 (0%)	1.000^§^
Surgical Approach, n (%)				0.168^§^
- Laparoscopic	44^#^ (88%)	31^#^ (83.8%)	13 (100%)	
- Robotic	6 (12%)	6 (16.2%)	0 (0%)	
Conversion rate (n, %)	0 (0%)	0 (0%)	0 (0%)	-
Multivisceral resection, n (%)	7 (14%)	6 (16.2%)	1 (7.7%)	0.660^§^
Cholecystectomy, n (%)	5 (10%)	4 (10.8%)	1 (7.7%)	1.000^§^
ICU LOS, days (IQR)	0 (0–0)	0 (0–0.5)	0 (0–0)	0.571^**†**^
Total LOS, days (IQR)	4 (3–6)	4 (3–5.5)	5 (4–10)	**0.047**^**†**^
Overall complications, n (%)	23 (46%)	18 (48.6%)	5 (38.5%)	0.747
Major complications, n (%)	7 (14%)	6 (16.2%)	1 (7.7%)	0.660^§^
IIIA	4 (8%)	4 (10.8%)	0 (0%)	
IIIB	1 (2%)	1 (2.7%)	0 (0%)	
IVA	1 (2%)	1 (2.7%)	0 (0%)	
IVB	1 (2%)	0 (0%)	1 (7.7%)	
Biochemical leak, n (%)	13 (26%)	10 (27%)	3 (23.1%)	1.000^§^
POPF, n (%)	12 (24%)	10 (27%)	2 (15.4%)	0.480^§^
B	12 (24%)	10 (27%)	2 (15.4%)	
C	0 (0%)	0 (0%)	0 (0%)	
PPH, n (%)	3 (6%)	3 (8.1%)	0 (0%)	0.558^§^
A	0 (0%)	0 (0%)	0 (0%)	
B	3 (6%)	3 (8.1%)	0 (0%)	
C	0 (0%)	0 (0%)	0 (0%)	
Delayed gastric emptying, n (%)	0 (0%)	0 (0%)	0 (0%)	-
Splenic vein occlusion, n (%)	1 (2%)	1 (2.7%)	0 (0%)	1.000^§^
Splenic infarction, n (%)	1 (2%)	0 (0%)	1 (7.7%)	0.260^§^
Reoperation, n (%)	2 (4%)	2 (5.4%)	0 (0%)	1.000^§^
Hospital readmission, n (%)	14 (28%)	13 (35.1%)	1 (7.7%)	0.078^§^
90-day mortality, n (%)	0 (0%)	0 (0%)	0 (0%)	-

Bold indicates *p*-value < 0.05 is considered statistically significant

*BMI* body mass index; *IQR *Interquartile range; *ASA* American Society of Anesthesiologists physical status classification; *PNET* pancreatic neuroendocrine tumour; *SD* standard deviation; *ICU* Intensive care unit; *LOS* length of stay; *POPF* postoperative pancreatic fistula; *PPH* post Pancreatectomy hemorrhage

Major complications defined as grade 3a or higher according to *Clavien Dindo* classification

^*^Pearson Chi-square, §Fisher’s Exact Test, †Mann–Whitney U Test

Grade B POPF was observed in 12 patients (24%), 10 (27%) in the Kimura group, 2 (15.4%) in the Warshaw group (p = 0.480).

Grade B PPH occurred in three patients (6%), all in the Kimura group. Hospital readmission was more common in the Kimura group but without a significant difference compared to the Warshaw group (35.1% vs 7.7% respectively, *p* = 0.078). No 90-day mortality occurred in both groups.

### Time period 2008–2014 versus 2015–2022

Analyses of outcomes between the time periods 2008–2014 and 2015–2022 are shown in Table 4. No differences were observed between the groups regarding age, sex, BMI, and ASA. All salvage Warshaw procedures occurred in the first time period (p < 0.001). Another significant difference was found in LOS, which decreased in the second period as compared to the first period (4 versus 5 days, p = 0.01). No differences were observed between the time periods regarding major complications, readmission, or reoperation.

**Table 4:** Comparison of outcomes between time periods 2008–2014 and 2015–2022

	2008–2014 (n = 26)	2015–2022 (n = 24)	*P*
Age, y (IQR)	70 (66.5–72)	67 (56–73)	0.068^**†**^
Female, n (%)	14 (53.8%)	10 (42%)	0.389*
BMI, Kg/m^2^ (IQR)	23.1 (21.1–27.7)	27.4 (22.2–32)	0.464^**†**^
ASA score, n (%)			0.359*
1	6 (23.1%)	2 (8.3%)	
2	15 (57.7%)	17 (70.8%)	
3	5 (19.2%)	5 (20.8%)	
Incidental diagnosis, n (%)	11/19 (57.9%)	19/24 (79.2%)	0.131*
Pathology, n (%)			
Cystic lesion	10 (38.5%)	11(45.8%)	0.011*
PNET	8 (30.8%)	13 (54.2%)	
Other	8 (30.8%)	0 (0%)	
Size, mm (mean ± Sd)	21.0 ± 14.7	21.7 ± 12.0	0.521^**†**^
Operative time, min (± SD)	195 ± 73	181 ± 70	0.263^**†**^
Intraoperative blood loss, mL (IQR)	150 (100–450)	100 (50–200)	0.078^**†**^
Intra- or post-operative blood transfusion, n (%)	0 (0%)	1 (4.2%)	0.480^§^
Surgical Approach, n (%)			
- Laparoscopic	26 (100%)	17 (70.8%)	**0.003**^§^
- Robotic	0 (0%)	7 (29.2%)	
Technique, n (%)			** < 0.001**^§^
- Vessel Preserving	13 (50%)	24 (100%)	
- Vessel Resecting	13 (50%)	0 (0%)	
Conversion rate (n, %)	0 (0%)	0 (0%)	**-**
Multivisceral resection, n (%)	4 (15.4%)	3 (12.5%)	1.000^§^
Cholecystectomy, n (%)	3 (11.5%)	2 (8.3%)	1.000^§^
ICU LOS, days (IQR)	0 (0–0)	0 (0–0.75)	0.778^**†**^
Total LOS, days (IQR)	5 (4–5.5)	4 (3–5)	**0.010**^**†**^
Overall complications, n (%)	12 (46.2%)	11 (45.8%)	1.000^§^
Major complications, n (%)	4 (15.4%)	3 (12.5%)	1.000^§^
IIIA	2 (7.7%)	2 (8.3%)	
IIIB	0 (0%)	1 (4.2%)	
IVA	1 (3.8%)	0 (0%)	
IVB	1 (3.8%)	0 (0%)	
Biochemical leak, n (%)	5 (19.2%)	8 (33.3%)	0.256*
POPF, n (%)	7 (26.9%)	5 (20.8%)	0.614*
B	7 (26.9%)	5 (20.8%)	
C	0 (0%)	0 (0%)	
PPH, n (%)	1 (3.8%)	2 (8.3%)	
A	0 (0%)	0 (0%)	0.602^§^
B	1 (3.8%)	2 (8.3%)	
C	0 (0%)	0 (0%)	
Delayed gastric emptying, n (%)	0 (0%)	0 (0%)	-
Splenic vein occlusion, n (%)	1 (3.8%)	0 (0%)	1.000^§^
Splenic infarction, n (%)	1 (3.8%)	0 (0%)	1.000^§^
Reoperation, n (%)	1 (3.8%)	1 (4.2%)	1.000^§^
Hospital readmission, n (%)	6 (23.1%)	8 (33.3%)	0.420*
90-day mortality, n (%)	0	0	-

Bold indicates *p*-value < 0.05 is considered statistically significant

*BMI* body mass index; *IQR *interquartile range; *ASA* American Society of Anesthesiologists physical status classification; *PNET* pancreatic neuroendocrine tumour; *SD* standard deviation; *ICU* intensive care unit; *LOS* length of stay; *POPF* postoperative pancreatic fistula; *PPH* post Pancreatectomy hemorrhage

Major complications defined as grade 3a or higher according to *Clavien Dindo* classification

^*^Pearson Chi-square, §Fisher’s Exact Test, †Mann–Whitney U Test

### LSPDP versus RSPDP

Analyses of outcomes between LSPDP and RSPDP are reported in Table 5. No significant differences were observed between both groups.

**Table 5:** Comparison of outcomes between laparoscopic and robotic spleen-preserving distal pancreatectomy

	Overall (n = 50)	Laparoscopic (n = 44)	Robotic (n = 6)	*P*
Age, y (IQR)	65 (42–72)	65 (41–72)	58 (42–77)	0.795^**†**^
Female, n (%)	24 (48%)	23 (52.3%)	1 (16.7%)	0.192^§^
BMI, Kg/m^2^ (IQR)	25.8 (22.2–32.0)	29.0 (22.0–33.7)	25.4 (23.5–30.7)	0.816^**†**^
ASA score, n (%) 1	8 (16%)	8 (18.2%)	0 (0%)	0.466*
2	32 (64%)	27 (61.4%)	5 (83.3%)	
3	10 (20%)	9 (20.5%)	1 (16.7%)	
Incidental diagnosis, n (%)	30 (69.8%)	24 (64.9%)	6 (100%)	0.082*
Pathology, n (%)				0.086
Cystic lesion	21 (42%)	20 (45.5%)	1 (16.7%)	*
PNET	21 (42%)	16 (36.4%)	5 (83.3%)	
Other	8 (16%)	8 (18.2%)	0 (0%)	
Size, mm (mean ± Sd)	26.0 ± 14.8	27.4 ± 15.8	19.3 ± 4.6	0.217^**†**^
Operative time, min (± SD)	185 ± 71	195 ± 70	214 ± 75.2	0.402^**†**^
Intraoperative blood loss, mL (IQR)	100 (50–262)	100 (65–287.5)	65 (45–150)	0.140^**†**^
Intra- or post-operative blood transfusion, n (%)	1 (2%)	1 (2.3%)	0 (0%)	1.000^§^
Technique, n (%)				0.319^§^
- Vessel Preserving	13 (26%)	13 (29.5%)	0 (0%)	
- Vessel Resecting	37 (74%)	31 (70.5%)	6 (100%)	
Conversion rate (n, %)	0 (0–0)	0	0	-
Multivisceral resection, n (%)	7 (14%)	6 (13.6%)	1 (16.7%)	1.000^§^
Cholecystectomy, n (%)	5 (10%)	5 (11.4%)	0 (0%)	1.000^§^
ICU LOS, days (IQR)	0 (0–0)	0 (0–1)	0.5 (0–1)	0.137†
Total LOS, days (IQR)	4 (3–6)	4.5 (3–7)	4 (3–4)	0.163^**†**^
Overall complications, n (%)	23 (46%)	20 (45.5%)	3 (50%)	1.000^§^
Major complications, n (%)	7 (14%)	5 (11.4%)	2 (33.3%)	0.192^§^
IIIA	4 (8%)	2 (4.5%)	2 (33.3%)	
IIIB	1 (2%)	1 (2.3%)	0 (0%)	
IVA	1 (2%)	1 (2.3%)	0 (0%)	
IVB	1 (2%)	1 (2.3%)	0 (0%)	
Biochemical leak, n (%)	13 (26%)	11 (25%)	2 (33.3%)	0.643^§^
POPF, n (%)	12 (24%)	10 (22.7%)	2 (33.3%)	0.621^§^
B	12 (24%)	10 (22.7%)	2 (33.3%)	
C	0 (0%)	0 (0%)	0 (0%)	
PPH, n (%)	3 (6%)	2 (4.5%)	1 (16.7%)	0.324^§^
A	0 (0%)	0 (0%)	0 (0%)	
B	3 (6%)	2 (4.5%)	1 (16.7%)	
C	0 (0%)	0 (0%)	0 (0%)	
Delayed gastric emptying, n (%)	0 (0%)	0 (0%)	0 (0%)	-
Splenic vein occlusion, n (%)	1 (2%)	1 (2.3%)	0 (0%)	1.000^§^
Splenic infarction, n (%)	1 (2%)	1 (2.3%)	0 (0%)	1.000^§^
Reoperation, n (%)	2 (4%)	2 (4.5%)	0	1.000^§^
Hospital readmission, n (%)	14 (28%)	12 (27.3%)	2 (33.3%)	1.000^§^
90-day mortality, n (%)	0	0	0	-

*BMI* body mass index; *IQR* interquartile range; *ASA* American Society of Anesthesiologists physical status classification; *PNET* pancreatic neuroendocrine tumour; *SD* standard deviation; *ICU* intensive care unit; *LOS* length of stay; *POPF* postoperative pancreatic fistula; *PPH* post Pancreatectomy hemorrhage

Major complications defined as grade 3a or higher according to *Clavien Dindo* classification

^*^Pearson Chi-square, §Fisher’s Exact Test, †Mann–Whitney U Test

## Discussion

This study described the techniques and outcomes of LSPDP and RSPDP performed by a single surgeon in two hospitals where this systematic approach was implemented.

Originally, the first reports on LDP described the techniques and outcomes of LDP including both malignant and benign pancreatic diseases [14, 24–27]. However, the surgical technique for benign and malignant lesions is profoundly different. For malignant lesions, distal pancreatectomy with splenectomy is usually indicated. In this oncological surgery, first described by Strasberg [28], and recently by Abu Hilal for the laparoscopic approach [29], radical resection of the whole left pancreas including Gerota’s fascia is performed to ensure extensive resection of all lymph nodes located along the splenic artery up to the splenic hilum [30].

Conversely, for benign lesions, a more parenchyma-sparing distal pancreatectomy should be preferred. Routine division at the pancreatic neck and splenectomy are in this case not mandatory. The pancreas is usually divided just proximally to the lesion, securing clear margins [29, 31]. Preservation of the spleen should be advised as it has been associated with a reduction of perioperative infections and long-term morbidity [2, 32]. Previous studies on LSPDP showed that LSPDP is a feasible and safe technique as it is associated with a similar short-term postoperative morbidity and recovery period compared to distal pancreatectomy with splenectomy [15, 33]. Nevertheless, SPDP should be attempted for benign or low-grade malignant lesions taking in consideration the long-term morbidity of post-splenectomy patients.

SPDP can be achieved by 2 different techniques. The first one described by *Warshaw* [11] in 1988, involves a segmental resection of both the splenic artery and vein while the gastroepiploic arcade and short gastric arteries are preserved to provide blood supply and drainage of the spleen. This approach may be associated with splenic infarction due to an inadequate inflow from gastric arteries, with consequent abscess. Another concern about this approach is the development of perigastric varices and hemorrhagic complications [34]. The other one, described by *Kimura* [10] in 1996, includes preservation of the splenic artery and vein. This seems to be a more anatomic and physiologic procedure with a lower risk of splenic infarction since the primitive spleen vascularization is maintained. However, this spleen-preserving technique is technically demanding as it requires a careful dissection of the pancreatic parenchyma from the splenic vein.

In the present study, a *Kimura* SPDP was always the intended procedure but could actually be achieved in 74% of the patients. In 13 patients (26%) a salvage Warshaw SPDP was required. Interestingly, all salvage Warshaw procedures were performed in the first time period, indicating that with increased surgical experience, a higher Kimura completion rate can be achieved.

In the present series, the overall complications rate of 46% (n = 23) was consistent with the literature and when analyzing only major complications, the incidence of 14% (n = 7) was lower, with no mortality registered [15, 35, 36]. The hospital LOS of 4 days, intraoperative blood loss of 100 mL, and operation time of 185 min were favorable when compared to other studies [7, 15, 33, 37, 38]. Only one patient required blood transfusion in the perioperative period. The hospital LOS in this study was lower compared to other and open series [37, 38], which is a recognized advantage of the laparoscopic and robot-assisted technique. Interestingly, complication rates and other postoperative outcomes remained stable over the two time periods, but only a reduced length of hospital stay was observed in the second period.

The reported conversion rate of 0% for both LSPDP and RSPDP highlights the importance of a methodical and experienced approach. Additionally, no significant differences in other postoperative outcomes were observed between LSPDP and RSPDP. However, although it did not reach significance, all salvage Warshaw procedures were performed in the LSPDP group in the first time period. In the second, no salvage Warshaw procedures were performed. This is even when we take into consideration our first 10 RSPDP. This may indicate that the learning curve in robot-assisted surgery may be very soft or even inexistent in centers with large previous laparoscopic experience.

The present study is limited by its retrospective nature and its small population. However, the analyzed data was derived from a prospectively maintained database of all consecutive LSPDP and RSPDP procedures performed by a single surgeon in two hospitals. Regarding the size of the study population, it should be kept in mind that even in a tertiary referral center, the patients for whom SPDP is indicated are limited.

## Conclusion

LSPDP and RSPDP are increasingly being adopted due to their promising results. The surgical step-by-step description of minimally invasive SPDP of the current study describes the tips and tricks we developed during our 14 years of experience, to standardize a reproducible and safe technique for the minimally invasive surgical treatment of distal pancreatic lesions where spleen preservation is indicated. In the authors’ experience, these steps are reproducible with either a laparoscopic or robot-assisted approach and can help to achieve optimal intra- and postoperative outcomes.

## Supplementary Information

Below is the link to the electronic supplementary material.

Supplementary file1 (MP4 93575 KB)

Supplementary file2 (MP4 96410 KB)

## Abbreviations

ASA: American society of Anesthesiologists physical status classification system
IPMN: Intraductal papillary mucinous neoplasm
ISGPS: International Study Group on Pancreatic Surgery
LOS: Length of stay
LDP: Laparoscopic distal pancreatectomy
LSPDP: Laparoscopic spleen-preserving distal pancreatectomy
MCN: Mucinous cystic neoplasm
PNET: Pancreatic neuroendocrine tumor
POPF: Postoperative pancreatic fistulas
PPH: Postpancreatectomy hemorrhage
RSPDP: Robotic spleen-preserving distal pancreatectomy
SPDP: Spleen-preserving distal pancreatectomy
